# Cancer risk in childhood-onset systemic lupus

**DOI:** 10.1186/ar4388

**Published:** 2013-11-22

**Authors:** Sasha Bernatsky, Ann E Clarke, Jeremy Labrecque, Emily von Scheven, Laura E Schanberg, Earl D Silverman, Hermine I Brunner, Kathleen A Haines, Randy Q Cron, Kathleen M O’Neil, Kiem Oen, Alan M Rosenberg, Ciarán M Duffy, Lawrence Joseph, Jennifer L Lee, Mruganka Kale, Elizabeth M Turnbull, Rosalind Ramsey-Goldman

**Affiliations:** 1 McGill University, 1020 Pine Avenue West, Montreal H3A 1A2, QC, Canada; 2 Research Institute of the McGill University Health Centre, 687 Pine Avenue West, V-Building, Montreal H3A 1A1, QC, Canada; 3 Divisions of Rheumatology and Clinical Epidemiology, McGill University Health Centre, 687 Pine Avenue West, V-Building, Montreal H3A 1A1, QC, Canada; 4 Department of Pediatric Rheum, University of California at San Francisco, 505 Parnassus Box 0107, San Francisco 94143, CA, USA; 5 Department of Pediatrics, Duke University Medical Center, PO Box 3212, Durham 27715-3212, NC, USA; 6 Division of Rheumatology, Hospital for Sick Children, 555 University Avenue, Toronto M5G 1X8, ON, Canada; 7 Rheumatology, Cincinnati Children's Hospital Medical Center, 3333 Burnet Ave, Cincinnati 45229, OH, USA; 8 Department of Pediatrics, Hackensack University Medical Center, 30 Prospect Ave, Hackensack 07601, NJ, USA; 9 Pediatric Rheumatology, University of Alabama-Birmingham, 1530 Third Ave South, SHEL 176, Birmingham 35294-2182, AL, USA; 10 Division of Pediatric Rheumatology, Riley Hospital for Children, 699 Riley Hospital Drive, Indianapolis 46202, IN, USA; 11 Pediatrics RR149 Rehab Ctr, University of Manitoba, 800 Sherbrook Street, Winnipeg R3A 1M4, MB, Canada; 12 Department of Pediatrics, Royal University Hospital, 103 Hospital Dr, Saskatoon S7N 0W8, SK, Canada; 13 Pediatrics, Children's Hospital of Eastern Ontario, 401 Smyth Rd, Ottawa K1H 8M5, ON, Canada; 14 Northwestern University Feinberg School of Medicine, McGaw Pavilion, 240 E. Huron Street, Suite M-300, Chicago 60611, IL, USA

## Abstract

**Introduction:**

The aim of this study was to assess cancer incidence in childhood-onset systemic lupus erythematosus (SLE).

**Methods:**

We ascertained cancers within SLE registries at 10 pediatric centers. Subjects were linked to cancer registries for the observational interval, spanning 1974 to 2009. The ratio of observed to expected cancers represents the standardized incidence ratio (SIR) or relative cancer risk in childhood-onset SLE, versus the general population.

**Results:**

There were 1020 patients aged <18 at cohort entry. Most (82%) were female and Caucasian; mean age at cohort entry was 12.6 years (standard deviation (SD) = 3.6). Subjects were observed for a total of 7,986 (average 7.8) patient-years. Within this interval, only three invasive cancers were expected. However, 14 invasive cancers occurred with an SIR of 4.7, 95% confidence interval (CI) 2.6 to 7.8. Three hematologic cancers were found (two non-Hodgkin’s lymphoma, one leukemia), for an SIR of 5.2 (95% CI 1.1 to 15.2). The SIRs stratified by age group and sex, were similar across these strata. There was a trend for highest cancer occurrence 10 to 19 years after SLE diagnosis.

**Conclusions:**

These results suggest an increased cancer risk in pediatric onset SLE versus the general population. In absolute terms, this represents relatively few events. Of note, risk may be highest only after patients have transferred to adult care.

## Introduction

In the past decade, there have been several large studies elucidating cancer risk in adults with systemic lupus erythematosus (SLE). However, relatively little is known about cancer risk in childhood-onset SLE. This knowledge gap is important since pediatric patients have a long disease duration and a heavy burden of severity. Moreover, extrapolations from adult-onset SLE populations are inappropriate, given the drastic clinical differences (in disease activity, treatment, organ involvement, and so forth) between pediatric-onset SLE and adult-onset SLE [1-3].

Our objective was to assess the observed cancer incidence in a large clinical cohort of pediatric-onset SLE patients. We compared the observed number of cancers over the observation interval with that which would be expected based on age-specific and sex-specific general population cancer incidence rates.

## Methods

We ascertained cancers within SLE registries at 10 pediatric centers, located in: Birmingham, AL, USA; Cincinnati, OH, USA; Durham, NC, USA; Hackensack, NJ, USA; Oklahoma, OK, USA; San Francisco, CA, USA; Montreal, QC, Canada; Toronto, ON, Canada; Saskatoon, SK, Canada; and Winnipeg, MB, Canada. Subjects were linked to state or provincial cancer registries for the observational interval, spanning 1974 to 2009.

Follow-up was calculated from the date first seen at the clinic with an SLE diagnosis and the first of three possible events: death, cancer, or end of study interval (December 2009). We pooled observed cancers and person-years of observation. The cancers expected to occur were calculated by multiplying the person-years in the cohort by the geographically matched age-specific, sex-specific, and calendar year-specific cancer rates, obtained from the same cancer registries that performed the cohort linkages. The ratio of observed to expected cancers represents the standardized incidence ratio (SIR), or the relative cancer risk in pediatric-onset SLE, versus the general population. *In situ* cancers occurring in the SLE cohort were excluded, since these lesions are generally not included in general population cancer rates.

We provided estimates for total cancer and for hematological cancers (the most common cancer type, and the one most often noted in prior case reports). We also present results stratified by sex, age group, and SLE duration.

Institutional Review Board ethics approval, including waiver of informed consent, was provided by the McGill University Health Centre Institutional Review Board.

## Results

There were 1,020 patients aged <18 at cohort entry. Most patients (82%) were female and Caucasian; the mean age at cohort entry was 12.6 years (standard deviation = 3.6). Subjects were observed for a total of 7,986 (average 7.8) patient-years. Within this observation interval, only three invasive cancers were expected; however, 14 invasive cancers occurred (SIR = 4.7, 95% confidence interval (CI) = 2.6, 7.8). The SIRs stratified by age group and sex were similar across strata (Table 1). There was a trend for highest cancer occurrence within the period 10 to 19 years after SLE diagnosis.

**Table 1:** All observed and expected cancers, along with standardized incidence ratio and 95% confidence interval

**All cancers**	**Observed**	**Expected**	**SIR**	**95% CI**
Stratified by sex				
Male	2.0	0.5	4.4	0.5, 16.0
Female	12.0	2.6	4.6	2.4, 8.1
Stratified by age group				
0 to 19 years	3.0	0.8	3.9	0.8, 11.4
20+ years	11.0	2.3	4.8	2.4, 8.6
Stratified by SLE duration				
< 1 year	2.0	0.1	15.2	1.8, 54.9
1 to 4 years	1.0	0.6	1.8	0.0, 9.9
5 to 9 years	1.0	0.6	1.7	0.0, 9.6
10 to 19 years	8.0	0.9	9.2	4.0, 18.2
20+ years	2.0	0.7	3.1	0.4, 11.1

CI, confidence interval; SIR, standardized incidence ratio; SLE, systemic lupus erythematosus.

At the time of cancer diagnosis, mean SLE duration was 12.3 years (range 2 months, 25.2 years). We performed a sensitivity analysis excluding cancers that occurred within the first year of SLE diagnosis (since in these cases the SLE-like manifestations may have actually represented a paraneoplastic syndrome); here, the overall cancer SIR was 3.0 (95% CI = 2.3, 7.8).

Three hematologic cancers were found (two non-Hodgkin’s lymphoma, one leukemia), for an SIR of 5.2 (95% CI = 1.1, 15.2). The ages of the SLE patients (one male, one female) at lymphoma diagnosis were 16 and 28 years, and the leukemia case was female and diagnosed at age 7. The leukemia case and one Non-Hodgkin’s lymphoma were reported within the first year of SLE diagnosis.

The precision of the CIs was less when the SIRs were generated by sex strata (hematological cancer SIR for males = 7.6, 95% CI = 0.2, 42.3; and for females = 4.5, 95% CI = 0.5, 16.2). Similarly wide CIs were seen with the SIR for hematological cancers for the age group 0 to 19 (SIR = 7.7, 95% CI = 0.9, 27.7) and for the age group 20+ (SIR = 3.2, 95% CI = 0.2, 17.5). The nonhematological cancers included one cancer each of the bladder, brain, breast, and thyroid, three head and neck cancers, and four unspecified cancers. The head and neck malignancy types included one cancer of the lingual tonsil and the other two were squamous cell carcinomas not otherwise specified.

## Discussion

This study compared the cancer risk in pediatric-onset SLE patients with age-specific and sex-specific regional general population cancer rates. These results suggest an increased cancer risk in pediatric-onset SLE versus the general population, which appeared to be driven in part by hematological malignancies. Of course, in absolute terms, this still translates into relatively few events (1.75 incident cancers per 1,000 person-years), which is somewhat reassuring. A limitation of this study is that patients may have developed cancer after relocating to another state or province, thus under-representing the true cancer incidence in this cohort. Another limitation is that we are unable to comment on the relative importance of SLE disease itself, versus therapeutic drugs. We are also unable to evaluate the effects of race/ethnicity, or of SLE clinical factors such as type of organ involvement and disease severity.

Our study adds substantially to the pre-existing scant literature regarding malignancies in pediatric-onset rheumatic diseases. There are a few published case reports of malignancy occurring after the onset of pediatric-onset SLE [4-6] and these four case reports all document hematological malignancies: Hodgkin’s lymphoma, mucosa-associated lymphoid tissue lymphoma, Burkitt’s lymphoma, and acute lymphoblastic leukemia (ALL). The first three cases mentioned occurred in teenagers after several years of SLE duration, while the fourth case, which was ALL, occurred in a 6 year old within the first year of SLE diagnosis. In addition, two additional case reports document the simultaneous presentation of pediatric-onset SLE (fulfilling ACR criteria) and ALL, at ages 3 and 7 [7,8].

This possibly suggests a bimodal pattern to hematological malignancies in pediatric-onset SLE. The first phase of this bimodal pattern would correspond to the scenario of a young pediatric-onset SLE patient presenting with pancytopenia, where leukemia (for example, ALL) is a possible consideration. The second part of this bimodal pattern of hematological cancer risk in pediatric-onset SLE seems to be related to lymphoma (both Hodgkin’s lymphoma and non-Hodgkin’s lymphoma) in older individuals. Alternatively, the first peak might actually represent, at least in part, paraneoplastic presentations masquerading as pediatric-onset SLE; in either case, clinicians should remain vigilant to the possibility of a disease like ALL in such a setting.

Ours is the first cohort study of the cancer experience of a large number of patients with pediatric-onset SLE, comparing the observed number of cancers with those expected (based on age, sex, and calendar-year appropriate rates for the geographically relevant general population). In terms of a comparison with what is known in adults, an increased hematologic cancer risk (especially Non-Hodgkin’s lymphoma) has also been demonstrated in adult-onset SLE. Interestingly, in adult-onset SLE the highest cancer risk, relative to the general population, occurs in the youngest age group (<45 years) [9].

There is currently also interest in malignancy risk for other pediatric-onset rheumatic diseases, including juvenile idiopathic arthritis. Using similar methods to the current study, we found rather different results in juvenile idiopathic arthritis, for overall cancer, with six cancers observed and seven expected, leading to an overall SIR that was not elevated (SIR = 0.8, 95% CI = 0.3, 1.8) [10]. However, all cancers were hematologic, including five leukemia cases. This potentially represents a signal for a potentially increased risk of hematological cancer in JIA, similar to what we present here in pediatric-onset SLE. The etiology of this potentially increased risk of hematological malignancies in juvenile idiopathic arthritis also remains to be elucidated.

## Conclusions

This study represents the most up-to-date results from our multicenter initiative to clarify baseline cancer risk in pediatric-onset SLE. There is a possible increased risk in overall cancer, which may be driven by hematologic cancer risk. Further work in progress will compare risk across geography, race/ethnicity, and disease subset.

## Abbreviations

ALL: Acute lymphoblastic leukemia; CI: Confidence interval; SIR: Standardized incidence ratio; SLE: Systemic lupus erythematosus.

## Competing interests

The authors declare that they have no competing interests.

## Authors’ contributions

SB, AEC, EvS, LES, EDS, HIB, KAH, RQC, KMO, KO, AMR, CMD, MK, and RR-G contributed to the study design, data collection, data analysis, and interpretation and preparation of results. JL contributed to data preparation, data analysis and interpretation and presentation of results. LJ contributed to statistical analysis and the interpretation and preparation of the results. JLL and EMT participated in the design and coordination, and helped to draft the manuscript. In addition, all authors revised the manuscript and approved the final version.
